# Alcohol Use Disorder With Metabolic Dysfunction Is Associated With Adverse Health Impacts in a United States Clinical Setting

**DOI:** 10.1111/adb.70128

**Published:** 2026-03-04

**Authors:** Alexandra C. Wagner, Jeesun Jung, Joshua Reitz, Tyler Perlstein, LaToya Sewell, Melanie L. Schwandt, Nancy Diazgranados, Josephin Wagner, Daniel B. Rosoff, Falk W. Lohoff

**Affiliations:** ^1^ Section on Clinical Genomics and Experimental Therapeutics, National Institute on Alcohol Abuse and Alcoholism, National Institutes of Health Bethesda Maryland USA; ^2^ Office of the Clinical Director National Institute on Alcohol Abuse and Alcoholism, National Institutes of Health Bethesda Maryland USA; ^3^ NIH Oxford‐Cambridge Scholars Program University of Oxford Oxford UK

**Keywords:** alcohol use disorder, liver dysfunction, liver enzyme, liver fibrosis, metabolic dysfunction, metabolic syndrome

## Abstract

The combined disease burden of excessive alcohol consumption and metabolic dysfunction (MD) is an escalating global concern. Although it is well established that both factors adversely impact health, the biological characteristics and comorbidities of their overlap remain understudied in the United States. The present study investigated whether concurrent MD and alcohol use disorder (AUD) is associated with worse liver‐related and psychiatric health. A total of 1220 participants were recruited through the Natural History Protocol at the National Institutes of Health (NIH) and categorized into the following four groups: healthy controls (HC), individuals with MD (metHC), individuals with current AUD without MD (AUD) and those with both current AUD and MD (metAUD). Sociodemographic and clinical biomarkers, liver injury indices (Fibrosis‐4 [FIB‐4], LiverRisk, NAFLD fibrosis score [NFS]), liver enzymes and inflammatory markers (GGT, AST, ALT, CRP), liver function tests (albumin, bilirubin, PT‐INR), psychiatric and substance use comorbidities as well as current smoking were assessed in the four groups using analysis of covariance (ANCOVA). In addition, the clinical biomarkers were compared across three groups: mild (< 3 MD criteria) and severe (≥ 3) metAUD, as well as AUD only. Liver enzymes, noninvasive liver fibrosis scores and liver function tests showed additive effects across metHC, AUD and metAUD compared to HC, with the largest effects in metAUD for GGT, AST, ALT, CRP, albumin, direct bilirubin, FIB‐4, LiverRisk and NFS (*p* < 0.001). Psychiatric disorders also exhibited the most significant association with metAUD (*p* < 0.001). Within AUD, greater MD severity was associated with higher GGT, ALT, CRP, NFS and any DSM anxiety disorders (*p* < 0.05). These findings suggest that MD in the context of AUD is associated with greater liver dysfunction and psychiatric burden, supporting MD‐targeted treatment strategies in clinical care for AUD.

## Graphical abstract

This study contributes to understanding the health impacts of co‐occurring alcohol use disorder (AUD) and metabolic dysfunction (MD). Using clinical data from the NIH, individuals with both AUD and MD (metAUD) had the strongest associations with greater hepatic injury and inflammation, a less favorable liver function test profile, higher fibrosis burden, and greater psychiatric comorbidities relative to those with MD alone or AUD alone. These findings highlight the need for targeted research and clinical care for co‐occurring AUD and MD.

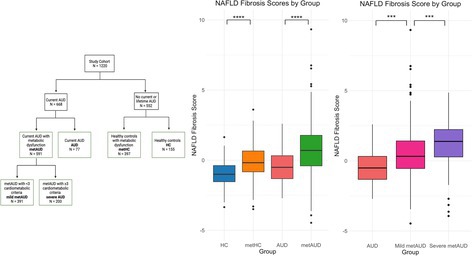

## 1 Introduction

Alcohol use disorder (AUD) is a leading global cause of morbidity and mortality, contributing approximately 7% of the disease burden and affecting over 1 billion people [1, 2]. Alcohol excess is a significant risk factor for chronic liver disease, which results in the approximately 2 million yearly liver‐related deaths worldwide [3, 4]. A 2023 Delphi consensus refined fatty liver disease nomenclature, adopting steatotic liver disease (SLD) as an umbrella term that includes metabolic dysfunction (MD)‐associated steatotic liver disease (MASLD), metabolic and alcohol‐associated liver disease (MetALD) and alcohol‐associated liver disease (ALD). With rising rates of obesity, type 2 diabetes (T2D) and heavy alcohol use, the global prevalence of both MASLD and ALD has steadily increased over the past few decades [5, 6].

Although obesity and alcohol consumption independently affect the liver, their combination has a partially additive and synergistic effect to significantly increase the risk of liver injury or mortality [7, 8, 9, 10]. Understanding the multifactorial impacts of AUD and MD is particularly important because of the limited treatment options, unclear guidance on safe alcohol consumption and the frequent late‐stage diagnosis of liver disease [8, 11, 12].

While previous research has summarized the prevalence of SLD in US population‐wide data [13, 14], limited insights exist regarding the impact of AUD and MD in clinical settings. Within this context, the primary objective of this study is to test whether concurrent MD and AUD (metAUD) is associated with greater liver dysfunction and greater psychiatric comorbidity than AUD alone in a clinical cohort at the National Institute on Alcohol Abuse and Alcoholism (NIAAA). The secondary objective is to examine whether greater MD severity in AUD patients is associated with worse health outcomes, as measured by liver fibrosis scores and function variables and psychiatric and substance use comorbidities. In this cross‐sectional observational study, we compared sociodemographic and clinical characteristics, three noninvasive liver fibrosis scores, alcohol consumption, psychiatric and substance use and current nicotine use and dependence among the following four groups: healthy controls (HC), healthy controls with MD (metHC), current AUD patients (AUD) and current AUD patients with MD (metAUD). Furthermore, we examined whether greater MD severity in AUD patients is associated with differences in liver fibrosis and function measures and in psychiatric and substance use comorbidities.

## 2 Materials and Methods

### 2.1 Study Participants

This cross‐sectional observation study enrolled 1359 participants between 21 January 2015 and 3 January 2024, under the NIAAA Natural History Protocol (14‐AA‐0181) at the National Institutes of Health (NIH). No longitudinal follow‐up was performed, and analyses used baseline assessments only. Eligibility criteria required participants to be at least 18 years old and either treatment‐seeking individuals in an inpatient alcohol treatment program or healthy volunteers from the community. Individuals were excluded if they were pregnant or breastfeeding, prisoners or had a severe medical or mental health disorder that would impair study participation. In our analytic sample, participants met one of two inclusion categories (i) current AUD within 12 months or (ii) neither lifetime nor current AUD for HCs. Consistent with this, we excluded participants who had AUD prior to the past 12 months but did not have current AUD (*n* = 139). The final analytic sample comprised 1220 individuals. The sample size was based on the number of eligible participants who completed the study by the time of data collection. All participants provided written, informed consent in accordance with the Declaration of Helsinki [15] and the NIAAA institutional review board.

### 2.2 Assessment of Clinical Measurements

Data collection took place at the NIH Clinical Center in Bethesda, MD, a US research hospital that delivers protocol‐driven care with uniform phenotyping at no cost to participants. Participants provided demographic information, fasted blood samples, vital signs and completed the Structured Clinical Interview for the Diagnostic and Statistical Manual (DSM)‐IV or ‐5 (SCID‐IV or SCID‐5) [16, 17]. Missing data were minimal, and no imputation was made. Liver measurements included the liver enzymes gamma‐glutamyl transferase (GGT), alanine aminotransferase (ALT), aspartate aminotransferase (AST), the systemic inflammation marker C‐reactive protein (CRP), liver function tests of albumin, total bilirubin, direct bilirubin and PT‐INR. Liver fibrosis was assessed using the noninvasive Fibrosis‐4 (FIB‐4), LiverRisk scores and nonalcoholic fatty liver disease (NAFLD) fibrosis score (NFS) [18, 19, 20]. FIB‐4 scores were categorized as < 1.3, 1.3–2.67 and > 2.67 [21]. LiverRisk scores, calculated using liverriskscore.com, were classified as minimal (< 6), low (6–10), medium (10–15) or high risk (> 15) [19]. The website provides a set range that can be input for each value, and if the actual value was outside of the predetermined range, the minimum or maximum value possible was used. NFS were classified using standard cutoffs: < −1.455 rules out advanced fibrosis and > 0.676 rules in advanced fibrosis, with intermediate values treated as indeterminate [18]. Transient elastography via the FibroScan was available for a subset of treatment‐seeking inpatients (*n* = 62) and provided liver stiffness and controlled attenuation parameter (CAP) scores as continuous measures. Given the limited availability, FibroScan‐based analyses were presented as exploratory.

Alcohol consumption variables were collected using the Alcohol Use Disorders Test (AUDIT) [22] and the 90‐day Timeline Followback (TLFB) [23]. The Brief Scale for Anxiety (BSA) [24], the Spielberger State–Trait Anxiety Inventory‐Y2 Score (STAI‐Y2) [25] and the Montgomery–Åsberg Depression Rating Scale (MADRS) [26] were collected as continuous measures of anxiety and depression. Lastly, the Fagerström Test for Nicotine Dependence (FTND) was administered to understand a participant's use and intensity of nicotine dependence [27]. Participants were classified into current smokers (*n* = 372) and nonsmokers (*n* = 835) based on status at admission/assessment. A small subset of participants (*n* = 13) did not complete the FTND, resulting in missing data for smoking status. The participants classified as current smokers were given a total FTND score from 0 to 10, with a higher number representing a more severe physical dependence on nicotine [27]. Participants not smoking at admission, which includes never‐smokers and ex‐smokers, were assigned a score of 0. FTND scoring and dependence severity calculations were conducted only among current smokers.

Psychiatric diagnoses and substance use disorders were based on lifetime and current diagnosis by the SCID‐IV/5 and the patient's medical history. Use of SCID‐IV versus SCID‐5 was determined by assessment date, with SCID‐5 implementation beginning in January 2017 (full transition completed by April 2017). Any mood disorder category includes patients with a medical history of depression and those found on the SCID to have persistent depression, major depression as well as bipolar I and bipolar II disorder. Any anxiety disorder category includes patients with a medical history of anxiety and those diagnosed on the SCID to have generalized anxiety, social anxiety, agoraphobia and/or panic disorder, as well as social or specific phobias. For substance use disorders, the stimulants category included current or past amphetamines, cocaine or stimulant use. The hallucinogens category encompassed current or past use of drugs classified as hallucinogens or phencyclidine (PCP). Categories for opioids, sedatives, inhalants and cannabis were restricted solely to current or past abuse or dependency of those specific substances.

### 2.3 Assessment of MD and AUD

Current AUD was determined using the SCID‐5 (*n* = 854). For participants assessed with the SCID‐IV (*n* = 366), a current AUD diagnosis was assigned if they met criteria for either alcohol abuse or dependence, given the excellent concordance between DSM‐IV and DSM‐5 criteria [28]. The final sample included 668 participants with current AUD and 552 HC with neither history of lifetime nor current AUD. MD was defined as having at least one of the following five cardiometabolic risk factors: body mass index (BMI) ≥ 25 kg/m^2^ (> 23 kg/m^2^ for Asian descent), fasting serum glucose ≥ 100 mg/dL, haemoglobin A1c (HbA1c) ≥ 5.7%, T2D diagnosis or medication, elevated blood pressure (130 mmHg systolic and/or 85 mmHg diastolic) or antihypertensive medication, plasma triglycerides ≥ 150 mg/dL or lipid‐lowering medication or high‐density lipoprotein (HDL) cholesterol (≤ 40 mg/dL for men and ≤ 50 mg/dL for women) or lipid‐lowering medication [29, 30].

Participants were categorized into one of four groups based on current AUD status and presence of MD criteria. The four participant groups were as follows: “metAUD” (current AUD with ≥ 1 MD criterion, *n* = 591), “AUD” (current AUD without MD, *n* = 77), “metHC” (≥ 1 MD criterion without AUD history, *n* = 397) and “HC” (healthy controls without AUD history and MD, *n* = 155).

To examine the impact of MD severity in AUD, current AUD participants were categorized based on the number of metabolic criteria met. Participants with < 3 MD criteria (*n* = 390) were classified as “mild metAUD,” while those meeting ≥ 3 MD criteria (*n* = 201) were classified as “severe metAUD.” This cutoff aligns with the diagnostic criteria for metabolic syndrome, which requires at least three cardiometabolic risk factors [29].

### 2.4 Statistical Analyses

All analyses were cross‐sectional and based on baseline measurements. Statistical analyses were performed between 6 September 2024 and 10 December 2024, using RStudio (Version 2024.04.2+764). Continuous variables were reported as means (SD) and categorical variables were presented as counts (%). Median (IQR) results are provided in the Supplemental Materials. Descriptive statistics were calculated for the HC versus metHC and AUD versus metAUD groups using *t*‐tests for continuous variables and χ^2^ or Fisher exact tests for categorical variables. Analysis of covariance (ANCOVA) was performed to examine associations of group differences in clinical and psychiatric measures, using the HC group as a reference group. Additionally, ANCOVA was used to investigate a relationship between clinical outcome and the severity of metAUD groups with the AUD reference group. All ANCOVA models adjusted for covariates of age, race and sex. All results were reported as an effect size (*b*), standard errors (SE) and *p*‐values. Clinical measures that did not meet normality assumptions (Shapiro–Wilk test, *p* < 0.05) had a natural log transformation applied before the analysis. As exploratory data with limited availability (*n* = 62), the transient elastography data (CAP score and liver stiffness) were evaluated with both mean [SD] and median (IQR). Significance was defined by a *p*‐value of < 0.05. Furthermore, a sensitivity analysis of FIB‐4 score was performed with participants with ages between 35 and 65 years to validate our finding of FIB‐4.

## 3 Results

### 3.1 Sample Characteristics

The final analytical sample included 1220 eligible participants (Figure S1), of whom 668 (54.8%) met criteria for current AUD, and 988 (81.0%) met at least one MD criterion. Mean (SD) age was 40.5 (13.5); 703 (57.6%) were male; 552 (45.2%) were White. Group classifications included the following: HC (*n* = 155, 12.7%), metHC (*n* = 397, 32.5%), AUD (*n* = 77, 6.3%) and metAUD (*n* = 591, 48.4%). 88% of all current AUD patients and 72% of HC with neither lifetime nor current AUD exhibit at least one metabolic risk factor, with metAUD patients more likely to exhibit hyperglycaemia, hypertension, hypertriglyceridemia and low HDL cholesterol compared to the metHC group (Table S1). Compared to the HC group, individuals in the metHC group were more likely to be male, older, Black, have lower educational attainment and report more days of heavy drinking. Compared to the AUD group, the metAUD group was more likely to be older and have higher alcohol consumption. Full descriptive statistics are in Table S2.

**FIGURE 1:**
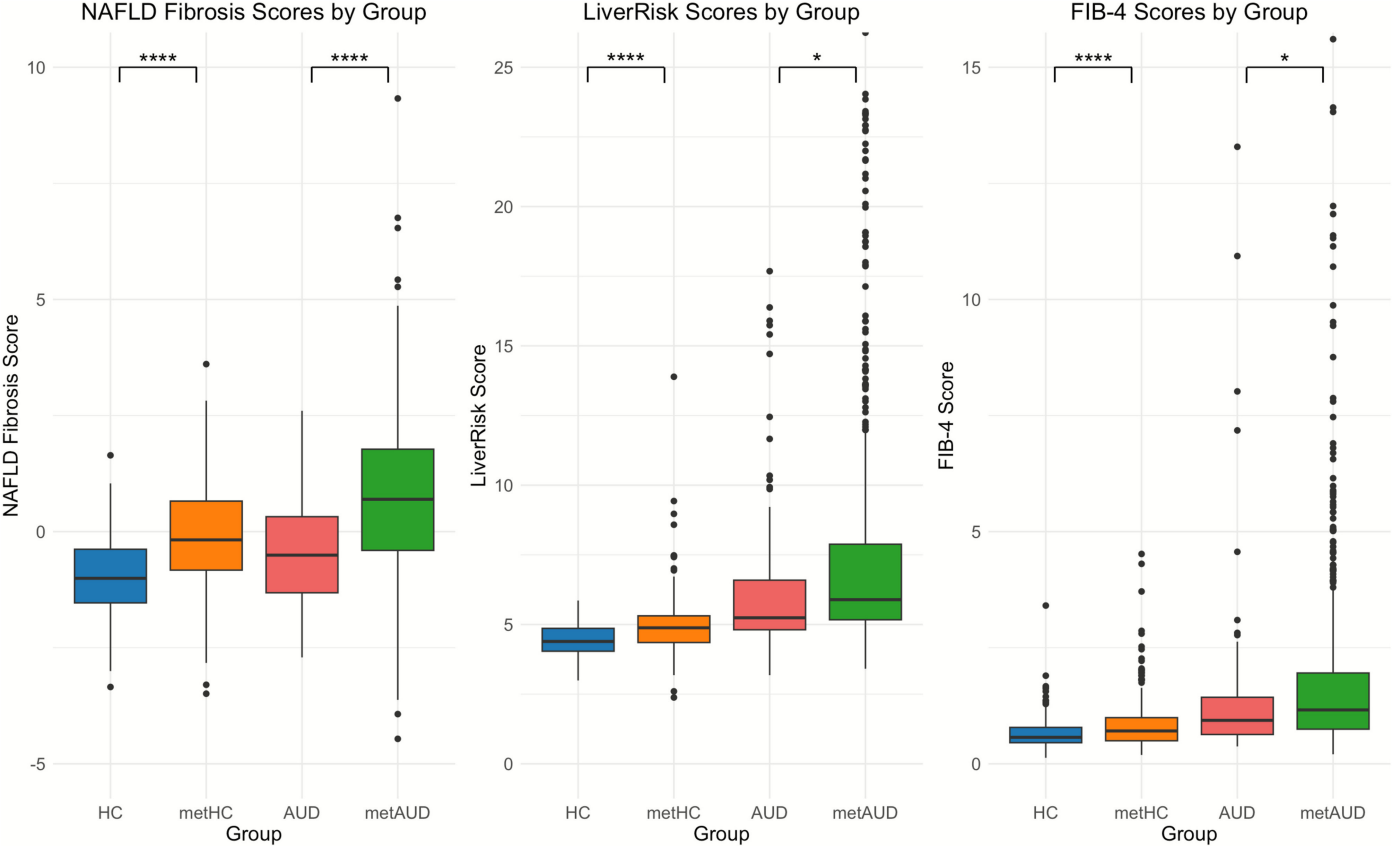
**Noninvasive liver fibrosis scores across HC, metHC, AUD and metAUD groups. (A)** NAFLD fibrosis scores by group. **(B)** LiverRisk scores by group. **(C)** FIB‐4 scores by group. Boxplots show median and interquartile ranges. Statistical analysis was conducted using *t* tests. Significance levels are indicated as follows: *p < 0.05, **p < 0.01, ***p < 0.001, ****p < 0.0001.

On clinical measures, the metAUD group showed significantly lower albumin and higher GGT, ALT, CRP and direct bilirubin than the AUD group (Table 1). The fibrosis indices FIB‐4, LiverRisk and NFS were also significantly higher in metAUD, consistent with a greater burden of advanced fibrosis (Table 1; Figure 1). When total bilirubin and the noninvasive fibrosis scores were analysed categorically, the same pattern persisted, with metAUD showing a higher proportion of participants in the advanced‐risk fibrosis categories based on standard FIB‐4 (> 2.67) and NFS (> 0.676) cutoffs and total bilirubin values (Table S3). Median and IQR values corresponding to the measures in Table 1 are provided in Table S4.

**TABLE 1:** Descriptive statistics by group, mean (SD).

	HC (*n* = 155)	metHC (*n* = 397)	*p*, HC vs. metHC	AUD (*n* = 77)	metAUD (*n* = 591)	*p*, AUD vs. metAUD
***Alcohol‐related characteristics***						
Total drinks (past 90 d)	38.8 (49.9)	45.5 (127.0)	0.38	663 (541)	1100 (4652)	0.03
No. of heavy drinking days (past 90 d)	0 (0)	0.4 (2.6)	0.003	8.1 (10.3)	13.8 (11.5)	< 0.001
AUDIT‐C score	9.1 (2.6)	2.6 (2.1)	0.31	9.1 (2.6)	9.7 (2.5)	0.047
***Metabolic criteria***						
BMI	22.1 (1.8)	28.4 (4.4)	< 0.001	21.9 (2.0)	27.9 (5.7)	< 0.001
Serum glucose, mg/dL	87.2 (6.1)	92.7 (12.6)	< 0.001	87.1 (6.8)	97.1 (17.5)	< 0.001
HbA1c, %	5.1 (0.3)	5.3 (0.4)	< 0.001	5.1 (0.4)	5.3 (0.7)	< 0.001
Systolic blood pressure, mm Hg	112.0 (8.9)	123.0 (14.7)	< 0.001	114 (9.0)	133 (16.3)	< 0.001
Diastolic blood pressure, mm Hg	68.2 (7.4)	73.7 (10.2)	< 0.001	71.3 (8.1)	84.5 (13.2)	< 0.001
Plasma triglycerides, mg/dL	67.3 (26.1)	87.0 (50.4)	< 0.001	72.9 (26.2)	108.0 (71.1)	< 0.001
LDL cholesterol, mg/dL	91.1 (28.6)	105.0 (32.2)	< 0.001	80.7 (32.5)	95.1 (36.1)	< 0.001
HDL cholesterol, mg/dL	69.6 (16.3)	58.0 (15.4)	< 0.001	81.0 (21.9)	68.4 (28.6)	< 0.001
Total cholesterol, mg/dL	174.0 (33.2)	180.0 (35.4)	0.06	176.0 (35.9)	184.0 (41.5)	0.08
***Chemistry***						
Platelet count, K/μL	256 (103)	249 (60.7)	0.41	227 (66.3)	221 (79.0)	0.46
GGT, U/L	17.3 (8.9)	25.5 (25.0)	< 0.001	99.5 (150.0)	139.0 (251.0)	0.048
AST, U/L	19.8 (5.6)	22.2 (14.1)	0.004	39.1 (38.3)	46.3 (53.4)	0.15
ALT, U/L	16.3 (9.4)	21.2 (15.0)	< 0.001	29.4 (25.1)	38.9 (40.4)	0.005
CRP, U/L	1.1 (1.5)	2.7 (4.3)	< 0.001	1.6 (2.8)	4.9 (14.9)	< 0.001
Albumin, g/dL	4.54 (0.27)	4.41 (0.29)	< 0.001	4.17 (0.48)	4.05 (0.47)	0.04
Total bilirubin, mg/dL	0.62 (0.37)	0.53 (0.28)	0.004	0.67 (0.32)	0.75 (0.68)	0.12
Direct bilirubin, mg/dL	0.15 (0.09)	0.14 (0.08)	0.22	0.18 (0.13)	0.23 (0.31)	0.004
PT‐INR	1.02 (0.07)	1.01 (0.08)	0.22	0.99 (0.09)	1.00 (0.10)	0.33
***Liver fibrosis scores***						
NAFLD fibrosis score	−1.07 (1.52)	−0.09 (1.18)	< 0.001	−0.39 (1.20)	0.77 (1.63)	< 0.001
LiverRisk score	4.46 (0.57)	4.9 (0.95)	< 0.001	6.6 (3.3)	7.4 (4.1)	0.031
FIB‐4 score	0.69 (0.4)	0.82 (0.5)	0.001	1.6 (2.2)	2.2 (4.1)	0.046
***Psychiatric scales***						
BSA	0.7 (1.4)	0.7 (1.8)	0.90	7.3 (7.7)	10.0 (8.2)	0.006
MADRS	1.0 (2.4)	0.9 (2.1)	0.53	10.1 (9.7)	13.5 (10.8)	0.005
STAI‐Y2	29.0 (7.6)	28.7 (7.6)	0.73	44.0 (13.0)	46.6 (13.3)	0.20
***Psychiatric disorders*** *						
Any DSM psychiatric disorder	14 (9.0%)	44 (11.1%)	0.58	45 (58.4%)	399 (67.5%)	0.15
Any DSM mood disorder	8 (5.2%)	33 (8.3%)	0.28	39 (50.6%)	311 (52.6%)	0.83
Any DSM anxiety disorder	2 (1.3%)	11 (2.8%)	0.47	20 (26.0%)	232 (39.3%)	0.03

*Denotes data presented as no. (%).

Abbreviations: ALT, alanine aminotransferase; AST, aspartate aminotransferase; AUD, alcohol use disorder; AUDIT, The Alcohol Use Disorders Test‐ Consumption; BMI, body mass index; BSA, Brief Scale for Anxiety; CRP, C‐reactive protein; DSM, Diagnostic and Statistical Manual of Mental Disorders; FIB‐4, Fibrosis‐4; GGT, gamma‐glutamyl transferase; HbA1c, haemoglobin A1c; HDL cholesterol, high density lipoprotein cholesterol; LDL cholesterol, low density lipoprotein cholesterol; MADRS, Montgomery–Åsberg Depression Rating Scale; NAFLD, nonalcoholic fatty liver disease; PT‐INR, prothrombin time‐international normalized ratio; STAI‐Y2, Spielberger State–Trait Anxiety Inventory‐Y2 Score.

The metAUD group had higher rates of comorbidities with DSM anxiety disorders, as well as higher BSA and MADRS scores than the AUD group (Table 1). Across all groups, the highest unadjusted percentages of individuals with depression (50.9%), anxiety (31.6%), posttraumatic stress disorder (PTSD) (26.7%), attention deficit hyperactivity disorder (ADHD) or attention deficit disorder (ADD) (13.0%), social or specific phobia (11.5%), agoraphobia and/or panic disorder (6.1%) and bipolar disorder (4.6%) were observed in the metAUD group. However, in pairwise contrasts, prevalences did not differ significantly between HC and metHC or between AUD and metAUD (Table S5). For substance use comorbidity, unadjusted prevalences of cannabis (49.2%), stimulant (33.2%), opioid (12.9%), hallucinogen (9.3%), sedative (5.8%) and inhalant (1.2%) use were also highest in the metAUD group, although none of the pairwise comparisons reached statistical significance (Table S6).

When the AUD group was stratified based on number of MD criteria, unadjusted percentages of individuals with depression (51.7%), anxiety (34.3%), PTSD (27.4%) and bipolar disorder (5.5%) were present in the metAUD group, although differences in disorder‐specific prevalences across severity groups were not statistically significant (Table S7).

### 3.2 Association of AUD and MD Using HCs as the Reference Group

In ANCOVA models using the HC reference group, liver enzymes GGT, AST and ALT and inflammatory marker CRP showed an additive effect from HC to metHC to AUD to metAUD, with the strongest effect sizes of GGT, AST, ALT and CRP in the metAUD group (Table 2). Among the liver function markers, albumin demonstrated the smallest effect size, while direct bilirubin exhibited the largest effect size in metAUD. Fibrosis indices FIB‐4, LiverRisk and NFS were likewise most significantly associated with metAUD, indicating a greater burden of advanced liver fibrosis. Our sensitivity analysis of FIB‐4 with participants aged between 35 and 65 also supported the strongest association with metAUD (Table S8).

**TABLE 2:** ANCOVA analysis of clinical variables, psychiatric comorbidities and substance use using healthy controls as a reference group.

	metHC (*n* = 397)			AUD (*n* = 77)			metAUD (*n* = 591)		
	Effect size (*b*)	St. error	*p*	Effect size (*b*)	St. error	*p*	Effect size (*b*)	St. error	*p*
***Chemistry***									
Platelet count, K/μL	−4.84	7.21	0.50	−22.80	10.50	0.03	−23.87	7.26	0.001
GGT, U/L	7.45	17.52	0.67	79.49	25.49	0.002	117.28	17.62	< 0.001
AST, U/L	3.74	3.82	0.33	19.94	5.56	< 0.001	26.90	3.84	< 0.001
ALT, U/L	5.53	2.89	0.056	12.79	4.20	0.002	21.69	2.91	< 0.001
CRP, U/L	1.37	1.08	0.21	0.22	1.55	0.89	3.12	1.09	0.004
Albumin, g/dL	−0.10	0.03	0.004	−0.34	0.05	< 0.001	−0.39	0.03	< 0.001
Total bilirubin, mg/dL	−0.9	0.03	0.008	0.04	0.05	0.38	0.03	0.03	0.37
Direct bilirubin, mg/dL	−0.007	0.02	0.55	0.03	0.02	0.053	0.05	0.02	< 0.001
PT‐INR	−0.01	0.007	0.08	−0.04	0.01	< 0.001	−0.03	0.007	< 0.001
***Liver fibrosis scores***									
NAFLD fibrosis score	0.54	0.11	< 0.001	0.20	0.16	0.22	0.97	0.11	< 0.001
LiverRisk score	0.24	0.29	0.42	1.82	0.43	< 0.001	2.49	0.29	< 0.001
FIB‐4 score	−0.01	0.28	0.98	0.73	0.41	0.075	1.11	0.28	< 0.001
***Psychiatric scales***									
BSA	0.29	0.59	0.62	6.89	0.86	< 0.001	9.39	0.59	< 0.001
MADRS	0.45	0.77	0.56	9.75	1.12	< 0.001	13.10	0.78	< 0.001
STAI‐Y2	1.47	1.05	0.16	16.85	1.53	< 0.001	19.45	1.06	< 0.001
***Psychiatric disorders***									
Any DSM psychiatric disorder	0.51	0.33	0.12	3.11	0.38	< 0.001	3.55	0.32	< 0.001
Any DSM mood disorder	0.75	0.41	0.07	3.32	0.44	< 0.001	3.45	0.39	< 0.001
Any DSM anxiety disorder	1.01	0.78	0.19	3.68	0.77	< 0.001	4.27	0.73	< 0.001

Abbreviations: ALT, alanine aminotransferase; AST, aspartate aminotransferase; AUD, alcohol use disorder; BSA, Brief Scale for Anxiety; CRP, C‐reactive protein; DSM, Diagnostic and Statistical Manual of Mental Disorders; FIB‐4, Fibrosis‐4; GGT, gamma‐glutamyl transferase; MADRS, Montgomery–Åsberg Depression Rating Scale; NAFLD, nonalcoholic fatty liver disease; PT‐INR, prothrombin time‐international normalized ratio; STAI‐Y2, Spielberger State–Trait Anxiety Inventory‐Y2 Score.

Psychiatric outcomes showed a similar graded pattern. The effect size of any DSM psychiatric, mood and anxiety disorder progressively increased across HC to metHC to AUD to metAUD, reaching the strongest association with metAUD compared to HC, as shown in Table 2. Symptom scales BSA, MADRS and STAI‐Y2 also demonstrated additive effects across metHC, AUD and metAUD, with the strongest association in metAUD. For substance‐use comorbidity, the AUD group without MD had the strongest association with DSM‐defined illicit drug use, cannabis use and nicotine use (Table S8).

### 3.3 Association of Clinical Variables by Metabolic Severity in AUD Patients

**FIGURE 2:**
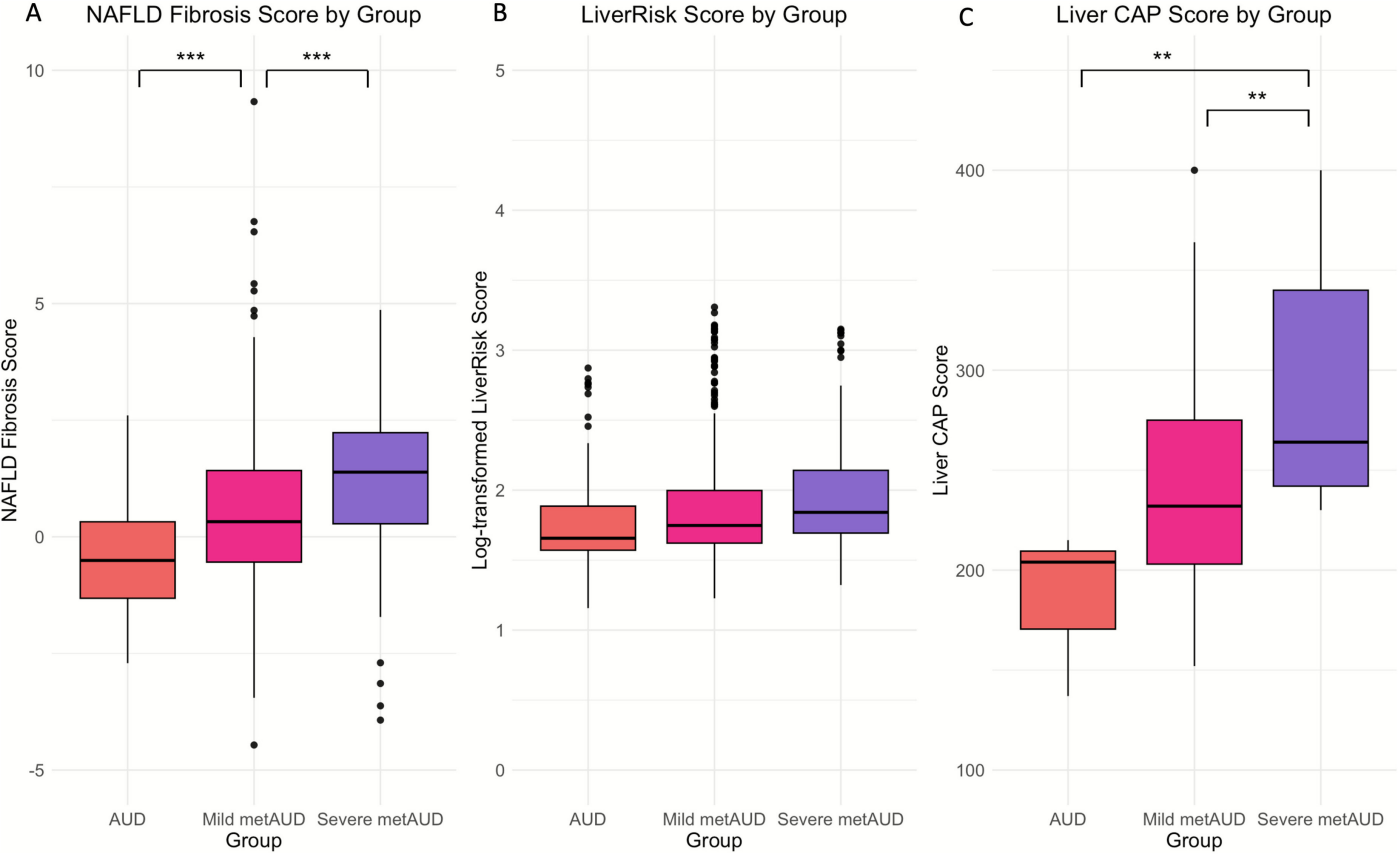
**Noninvasive liver fibrosis and steatosis measures across AUD, mild metAUD and severe metAUD groups. (A)** NAFLD fibrosis score by group. **(B)** LiverRisk score by group. **(C)** CAP score by group. Boxplots show median and interquartile ranges. Statistical analysis was conducted using *t* tests. Significance levels are indicated as follows: *p < 0.05, **p < 0.01, ***p < 0.001, ****p < 0.0001. Liver CAP Scores were calculated using a subset of inpatients with FibroScan data (AUD *n* = 3; mild metAUD *n* = 42; severe metAUD *n* = 17).

In ANCOVA models with the AUD reference group, natural log‐transformed GGT, AST, ALT and CRP showed approximately twofold larger effect sizes in the severe metAUD group compared to the mild metAUD group (Table 3; Figure S2). Albumin showed a graded decrease that did not reach statistical significance. The degree of association of NFS also increased with severity, with the largest effect size in the severe metAUD group (Table 3; Figure 2).

**TABLE 3:** ANCOVA analysis of clinical variables by metabolic severity in AUD patients.

	Mild metAUD (*n* = 390)			Severe metAUD (*n* = 201)		
	Effect size (*b*)	St. error	*p*	Effect size (*b*)	St. error	*p*
***Chemistry***						
Platelet count, K/μL	−2.78	9.58	0.77	4.10	10.43	0.69
GGT,^a^ U/L	0.22	0.14	0.13	0.48	0.16	0.002
AST,^a^ U/L	0.09	0.09	0.31	0.06	0.09	0.53
ALT,^a^ U/L	0.16	0.09	0.08	0.32	0.10	< 0.001
CRP,^a^ U/L	0.61	0.15	< 0.001	1.08	0.17	< 0.001
Albumin, g/dL	−0.03	0.05	0.57	−0.05	0.06	0.38
Total bilirubin, mg/dL	0.03	0.05	0.53	−0.06	0.06	0.35
Direct bilirubin, mg/dL	0.03	0.02	0.11	−0.02	0.02	0.43
PT‐INR	0.0002	0.01	0.98	0.01	0.01	0.18
***Liver fibrosis scores***						
NAFLD score	0.59	0.17	< 0.001	1.16	0.18	< 0.001
Liver risk score^a^	0.05	0.05	0.35	0.10	0.05	0.052
FIB‐4 score^a^	0.05	0.10	0.59	−0.10	0.10	0.32
***Psychiatric scales***						
BSA^a^	0.12	0.12	0.33	0.27	0.13	0.038
MADRS^a^	0.17	0.12	0.15	0.28	0.13	0.029
STAI‐Y2^a^	0.03	0.04	0.43	0.04	0.04	0.36
***Psychiatric disorders***						
Any DSM psychiatric disorder	0.41	0.27	0.13	0.39	0.30	0.18
Any DSM mood disorder	0.12	0.26	0.63	0.08	0.28	0.78
Any DSM anxiety disorder	0.63	0.29	0.03	0.67	0.31	0.03
***Nicotine use***						
Current smoker	−0.23	0.26	0.38	−0.58	0.28	0.04
FTND score	0.22	0.12	0.076	0.19	0.14	0.16

Abbreviations: ALT, alanine aminotransferase; AST, aspartate aminotransferase; AUD, alcohol use disorder; BSA, Brief Scale for Anxiety; CRP, C‐reactive protein; DSM, Diagnostic and Statistical Manual of Mental Disorders; FIB‐4, Fibrosis‐4; FTND, Fagerström Test for Nicotine Dependence; GGT, gamma‐glutamyl transferase; MADRS, Montgomery–Åsberg Depression Rating Scale; NAFLD, nonalcoholic fatty liver disease; PT‐INR, prothrombin time‐international normalized ratio; STAI‐Y2, Spielberger State–Trait Anxiety Inventory‐Y2 Score.

^a^ Represents where logarithmic adjustment was used.

Across psychiatric measures, patients with more severe MD exhibited greater psychiatric burden. Mean log‐transformed BSA and MADRS scores were most significantly associated with the severe metAUD group (Table 3). Additionally, the effect size of any DSM anxiety disorder was statistically higher in mild metAUD and severe metAUD compared to the AUD group (Table 3). However, current smoking showed a statistically lower effect size in the severe metAUD group relative to the AUD group.

### 3.4 Study in Transient Elastography by Metabolic Severity in AUD Patients

Among a subset of 62 participants with FibroScan data (AUD *n* = 3; mild metAUD *n* = 42; severe metAUD *n* = 17), MD severity was statistically associated with increased CAP score (Table S9; Figure 2). Furthermore, advanced liver stiffness was moderately associated with MD severity (AUD vs. mild metAUD, *p* = 0.04; AUD vs. severe metAUD, *p* = 0.056) (Figure S3). Median (IQR) values showed the same direction of effect (Table S9). The exploratory results support our primary findings of liver dysfunction and potential liver fibrosis with severe MD among AUD, although cautious interpretation is needed given the small sample size.

## 4 Discussion

The purpose of this study was to examine MD characteristics in individuals with AUD and evaluate associations of comorbid MD in AUD with liver enzymes and function tests, a systemic inflammatory marker, noninvasive fibrosis scores and psychiatric and substance use comorbidities in a US clinical cross‐sectional cohort. MD among AUD showed the strongest association with hepatic injury enzymes (GGT, AST, ALT) and CRP, albumin, direct bilirubin and noninvasive liver fibrosis scores (FIB‐4, LiverRisk, NFS) compared to MD alone or AUD alone. Taken together, this pattern is consistent with greater hepatic injury and inflammation, a less favourable liver function test profile and higher fibrosis burden in metAUD relative to the other groups. Additionally, the metAUD group demonstrated the most severe anxiety and depression and the highest rates of comorbid psychiatric disorders: anxiety, depression, PTSD, ADHD/ADD, agoraphobia, panic disorder and social or specific phobias. Notably, more advanced MD in AUD exhibited stronger associations with liver dysfunction and fibrosis scores than mild metAUD. Exploratory FibroScan data supported that the more severe metAUD group was associated with increased CAP score, although this subset should be interpreted with caution. Taken together, having MD in AUD showed the strongest association with greater hepatic injury and inflammation, a less favourable liver function test profile, higher fibrosis burden and greater psychiatric comorbidities relative to the MD alone or AUD alone.

### 4.1 Clinical Implications

The harm from excess alcohol consumption on the body is multifactorial, and no safe limit for alcohol intake has been established. Extensive research has linked heavy alcohol consumption to an increased risk of cardiovascular disease, obesity, hypertension, T2D, hypertriglyceridemia, stroke, certain cancers and other related disorders [31, 32, 33, 34], with frequent sex differences reported. AUD and MD often coexist, with higher rates of hypertension and T2D observed in individuals with AUD [35]. In > 50 000 hospitalized patients with T2D, AUD contributed to 55% of liver disease burden, while obesity and metabolic syndrome were attributed to < 10% of complications [36]. Similarly, a prospective analysis of 446 patients with a history of excessive alcohol consumption showed a stepwise increase in the risk of hepatic decompensation and mortality across diagnoses from MASLD to MetALD to ALD [37]. Across multiple large population cohorts, MASLD generally does not confer elevated mortality risk, whereas MetALD consistently shows higher all‐cause, liver‐related and cardiovascular mortality, with the highest risks observed among individuals with ALD and coexisting MD [38]. Given the high prevalence of MD in individuals with AUD, routine MD screening at the time of AUD evaluation using standard clinical measures such as blood pressure, BMI, lipid panels and fasting glucose, coupled with long‐term management strategies including lifestyle modification or targeted pharmacotherapy, may help mitigate risks of metabolic complications and progressive liver disease.

Semaglutide, a glucagon‐like peptide‐1 receptor (GLP‐1R) agonist, has been shown to reduce the risk of incidence and recurrence of AUD in patients with obesity or T2D [39], as well as decrease the risk of AUD‐related hospitalization in those with these comorbid conditions [40]. Tirzepatide, a dual GLP‐1R/glucose‐dependent insulinotropic polypeptide (GIPR) agonist, has also been associated with reduced alcohol consumption in observational analyses using social media text and selected patient follow‐up [41], although formal clinical trials evaluating dual GLP‐1R/GIPR agonists for AUD are still needed. Despite these promising signals, the biological mechanisms linking MD and AUD remain poorly understood [42], particularly at the genetic and molecular levels [43, 44]. These conditions likely require a multidisciplinary treatment strategy, and clinical trials specifically targeting comorbid AUD and MD are urgently needed to develop effective joint interventions.

Previous research also showed a higher prevalence of MD in individuals with bipolar disorder, schizophrenia, major depressive disorder and comorbid AUD, driven in part by psychotropic medication use and lifestyle factors [45, 46]. Supporting this, a worldwide survey showed that specific mental disorders were associated with a higher onset rate of physical conditions such as heart disease, hypertension and T2D [47]. Despite this elevated risk, MASLD and MASH are often overlooked in psychiatric care because they are frequently asymptomatic and not routinely assessed, leading to substantial underdiagnosis [48]. Given no sufficient pathological studies of psychiatric and substance use comorbidities seen in metAUD patients, further research to understand underlying mechanisms and to develop targeted treatment strategies is needed.

### 4.2 Strengths and Limitations

The presented research offers strengths that can inform future research and clinical care practices. First, diagnoses for AUD, psychiatric disorders and substance use disorders were conducted using the SCID‐IV or SCID‐5, which ensures validity and consistency across participants. Additionally, this paper includes a broad range of clinical and psychiatric measures which allow an in‐depth analysis of multiple comorbidities and their characteristics, rather than limiting the focus to the relationship between AUD and a single comorbidity. Second, the diversity of our clinical sample in terms of race and other sociodemographic factors enhances the generalizability of the findings towards minority groups and those who historically have had less access to treatment. In the United States, Black individuals comprise about 13% of the population, but in the metHC, AUD and metAUD groups, they represent about 42.8%, 44.2% and 42.1%, respectively (Table S1). Additionally, because patients at the NIH Clinical Center receive free care, our sample may be less impacted by a participant's financial constraints which can skew the data towards older individuals and those with health insurance. Third, our clinical sample is sufficiently large enough to provide adequate statistical power for examining the characteristics of MD in AUD.

The current findings should also be interpreted within the context of study limitations. Given the cross‐sectional nature of the data, it is important to note that the present findings have limited implications for understanding the bidirectional and causal relationships between AUD and MD. Future research should utilize longitudinal clinical follow‐up data to explore causal relationships and the temporal impacts between AUD and MD. The reliance on clinical samples also introduces the possibility of selection bias, because those accessing a clinical setting may have more severe AUD, greater health complications or higher levels of comorbid psychiatric or medication conditions compared to the general population. Furthermore, although the study examined the additive effects of MD and AUD, it was not able to account for the additive or interactive effects of coexisting psychiatric disorders and other substance use disorders within individuals, which frequently co‐occur in clinical populations.

The FIB‐4, LiverRisk scores and NFS have certain limitations that make them less accurate compared to transient elastography or enhanced liver fibrosis tests in predicting liver stages among individuals with MD in AUD [49, 50]. One notable limitation of the LiverRisk score is that it was derived and validated using an almost exclusively European cohort [19] and the NFS sample was 90% Caucasian [18]. In contrast, this study applied these tools to a clinical cohort with approximately 40% Black participants. While these blood‐based liver scores are less precise than other diagnostic measures unavailable in this dataset, they remain valuable tools for stratifying patients into risk categories.

## 5 Conclusions

In conclusion, having MD in AUD had stronger associations with alcohol consumption, liver‐related clinical measures and psychiatric comorbidities than individuals with AUD alone or MD alone. In addition, we found that greater MD severity in AUD was more strongly related to worsening liver enzymes, more potentially advanced fibrosis and greater psychiatric comorbidities, supporting the clinical need to address MD in individuals with AUD. Further studies are warranted to understand the longitudinal effects of MD on AUD as well as biological mechanisms of comorbidities of MD with AUD using multiomics data. Finally, our findings highlight the potential importance of interventions aimed at reducing MD in AUD to improve liver disease progression.

## Author Contributions

**Alexandra C. Wagner:** writing – original draft preparation, writing – review and editing, formal analysis. **Jeesun Jung:** writing – original draft preparation, writing – review and editing, methodology. **Joshua Reitz:** writing – review and editing. **Tyler Perlstein:** writing – review and editing. **LaToya Sewell:** investigation. **Melanie L. Schwandt:** resources. **Nancy Diazgranados:** funding acquisition, supervision. **Josephin Wagner:** writing – review and editing. **Daniel B. Rosoff:** writing – review and editing. **Falk W. Lohoff:** writing – review and editing, funding acquisition, supervision.

## Funding

This work was supported by the National Institutes of Health (NIH) intramural funding (ZIA‐AA000242 to F.W.L.) as part of the DICBR of the NIAAA. The NIAAA Natural History protocol was supported by the NIH intramural funding (ZIA‐AA000130 to N.D.) as part of the DICBR of the NIAAA. The funding source did not have any role in the design, collection, management, analysis and interpretation of the data. The preparation, review and decision to submit the manuscript for publication was made solely by the authors. The contributions of the NIH author(s) are considered Works of the United States Government. The findings and conclusions presented in this paper are those of the author(s) and do not necessarily reflect the views of the NIH or the US Department of Health and Human Services.

## Conflicts of Interest

The authors declare no conflicts of interest.

## Supporting information


**Figure S1:** Flowchart for developing sample groups in metAUD research.
**Table S1:** Distribution of metabolic dysfunction criteria in metHC and metAUD groups.
**Table S2:** Demographic characteristics by group.
**Table S3:** Categorical analysis of total bilirubin and noninvasive liver fibrosis scores.
**Table S4:** Median and IQR analysis of continuous variables shown in Table 1.
**Table S5:** Prevalence of psychiatric disorders by group.
**Table S6:** Prevalence of substance use disorders by group.
**Figure S2:** Liver enzymes and CRP values by metabolic severity subgroups in AUD.
**Table S7:** ANCOVA analysis of substance use and nicotine use.
**Table S8:** Sensitivity analysis of FIB‐4 scores using ages 35–65.
**Table S9:** Prevalence of psychiatric disorders by metabolic dysfunction severity in AUD.
**Table S10:** FibroScan analysis.
**Figure S3:** Exploratory analysis of FibroScan liver stiffness scores by metabolic severity subgroups in AUD.

## Data Availability

The data that support the findings of this study are available on request from the corresponding author. The data are not publicly available due to privacy or ethical restrictions.
