# Supplementary material for: Alcohol Use Disorder With Metabolic Dysfunction Is Associated With Adverse Health Impacts in a United States Clinical Setting
Source: Addict Biol. 2026 Mar 4;31(3):e70128. doi: 10.1111/adb.70128 (PMC12959954; doi:10.1111/adb.70128)
Supplement: Supplementary file 1 — Figure S1: Flowchart for developing sample groups in metAUD research. Table S1: Distribution of metabolic dysfunction criteria in metHC and metAUD groups. Table S2: Demographic characteristics by group. Table S3: Categorical analysis of total bilirubin and noninvasive liver fibrosis scores. Table S4: Median and IQR analysis of continuous variables shown in Table 1. Table S5: Prevalence of psychiatric disorders by group. Table S6: Prevalence of substance use disorders by group. Figure S2: Liver enzymes and CRP values by metabolic severity subgroups in AUD. Table S7: ANCOVA analysis of substance use and nicotine use. Table S8: Sensitivity analysis of FIB‐4 scores using ages 35–65. Table S9: Prevalence of psychiatric disorders by metabolic dysfunction severity in AUD. Table S10: FibroScan analysis. Figure S3: Exploratory analysis of FibroScan liver stiffness scores by metabolic severity subgroups in AUD. [file ADB-31-e70128-s001.docx]

**SUPPLEMENTAL MATERIALS**

**Figure S1:** Flowchart for Developing Sample Groups in metAUD research

**Table S1:** Distribution of Metabolic Dysfunction Criteria in metHC and metAUD groups

**Table S2:** Demographic Characteristics by Group

**Table S3:** Categorical Analysis of Total Bilirubin and Non-invasive Liver Fibrosis Scores

**Table S4:** Median and IQR Analysis of Continuous Variables Shown in Table 1

**Table S5:** Prevalence of Psychiatric Disorders by Group

**Table S6:** Prevalence of Substance Use Disorders by Group

**Figure S2:** Liver enzymes and CRP values by metabolic severity subgroups in AUD

**Table S7:** ANCOVA Analysis of Substance Use and Nicotine Use

**Table S8:** Sensitivity Analysis of FIB-4 Scores using Ages 35-65

**Table S9:** Prevalence of Psychiatric Disorders by Metabolic Dysfunction Severity in AUD

**Table S10:** FibroScan Analysis

**Figure S3:** Exploratory analysis of FibroScan liver stiffness scores by metabolic severity subgroups in AUD

**Figure S1: Flowchart for Developing Sample Groups in metAUD research**

**Table S1:** **Distribution of Metabolic Dysfunction Criteria in metHC and metAUD groups** No. (%)

|  | **metHC**  **(n=397)** | **metAUD**  **(n=591)** | **P-value** |
| --- | --- | --- | --- |
| BMI ≥ 25 [>23 for Asian descent], | 330 (83.1%) | 424 (71.7%) | < 0.001 |
| Hyperglycemia (Fasting serum glucose ≥ 100 mg/dL, HbA1c ≥ 5.6 %, T2D, or treatment for T2D) | 148 (37.3%) | 299 (50.6%) | < 0.001 |
| Hypertension (Blood Pressure ≥ 130/85 mmHg or Antihypertensive Drug Treatment | 143 (36.0%) | 403 (68.5%) | < 0.001 |
| Hypertriglyceridemia (Plasma Triglycerides ≥ 150 mg/dL or lipid lowering treatment) | 39 (9.8%) | 101 (17.1%) | 0.002 |
| Plasma HDL Cholesterol (≤ 40 mg/dL (male); ≤ 50 mg/dL (female)) | 80 (20.2%) | 84 (14.2%) | 0.02 |
| Note: AUD; alcohol use disorder, BMI; body mass index, HbA1c; hemoglobin A1c, T2D; type 2 diabetes, HDL; high density lipoprotein | | | |

**Table S2:** **Demographic Characteristics by Group,** No. (%)

|  | **HC**  **(n=155)** | **metHC**  **(n=397)** | **P-Value**  HC vs metHC | **AUD**  **(n=77)** | **metAUD (n=591)** | **P-Value**  AUD vs metAUD |
| --- | --- | --- | --- | --- | --- | --- |
| ***Sociodemographic***  ***characteristics*** | | | | | | |
| Sex | | | | | | |
| Male | 52 (33.5%) | 201 (50.6%) | <0.001 | 45 (58.4%) | 405 (68.5%) | 0.08 |
| Female | 103 (66.5%) | 196 (49.4%) |  | 32 (41.6%) | 186 (31.5%) |  |
| Age^**^, y | 32.0 (11.7) | 38.2 (14.0) | <0.001 | 38.3 (11.4) | 44.6 (12.4) | <0.001 |
| Race | | | | | | |
| White | 97 (62.6%) | 157 (39.5%) | <0.001 | 35 (45.5%) | 263 (44.5%) | 0.90 |
| Black | 37 (23.9%) | 170 (42.8%) |  | 34 (44.2%) | 249 (42.1%) |  |
| Asian | 14 (9.0%) | 38 (9.6%) |  | 1 (1.3%) | 15 (2.5%) |  |
| American Indian/  Alaska Native | 0 (0%) | 2 (0.5%) |  | 0 (0%) | 8 (1.4%) |  |
| Multiracial | 5 (3.2%) | 12 (3.0%) |  | 3 (3.9%) | 22 (3.7%) |  |
| Other Race | 2 (1.3%) | 18 (4.5%) |  | 4 (5.2%) | 34 (5.8%) |  |
| Annual Household Income, ($/y) | | | | | | |
| < $20,000 | 24 (15.5%) | 52 (13.1%) | 0.50 | 27 (35.1%) | 221 (37.4%) | 0.43 |
| $20,000 – $74,999 | 78 (50.3%) | 183 (46.1%) |  | 37 (48.1%) | 239 (40.4%) |  |
| > $74,999 | 53 (34.2%) | 153 (38.5%) |  | 13 (16.9%) | 126 (21.3%) |  |
| Education^**^, y | 16.7 (2.6) | 15.9 (3.3) | 0.001 | 14.2 (2.6) | 13.9 (2.9) | 0.42 |
| ***Nicotine Use*** |  |  |  |  |  |  |
| Current Smoker | 3 (1.9%) | 23 (5.8%) | 0.09 | 45 (58.4%) | 301 (50.9%) | 0.3 |
| FTND Score^**^ | 1.7 (1.5) | 2.7 (2.7) | 0.4 | 3.0 (2.4) | 3.8 (2.4) | 1 |
| Note: AUD; alcohol use disorder, FTND; Fagerström Test for Nicotine Dependence. ^**^Denotes data presented as mean (SD). | | | | | | |

**Table S3: Categorical Analysis of Total Bilirubin and Non-invasive Liver Fibrosis Scores,** No. (%)

|  | **HC**  **(n= 155)** | **metHC (n= 397)** | **P-Value**  HC vs metHC | **AUD**  **(n=77)** | **metAUD (n=591)** | **P-Value**  AUD vs metAUD |
| --- | --- | --- | --- | --- | --- | --- |
| ***Total Bilirubin,*** mg/dL | | | | | | |
| < 1.3 | 146 (94.2%) | 387 (98.0%) | 0.04 | 74 (96.1%) | 520 (88.0%) | 0.03 |
| ≥ 1.3 | 9 (5.8%) | 8 (2.0%) |  | 3 (3.9%) | 71 (12.0%) |  |
| ***NAFLD Fibrosis Score*** | | | | | | |
| < -1.455 | 44 (28.9%) | 42 (10.7%) | <0.001 | 15 (19.5%) | 36 (6.1%) | <0.001 |
| -1.455 – 0.676 | 102 (67.1%) | 253 (64.5%) |  | 49 (63.6%) | 255 (43.4%) |  |
| > 0.676 | 6 (4.0%) | 97 (24.7%) |  | 13 (16.9%) | 296 (50.4%) |  |
| ***LiverRisk Score*** | | | | | | |
| < 6 | 155 (100%) | 366 (92.2%) | <0.001 | 54 (70.1%) | 307 (51.9%) | 0.02 |
| 6 ~ 10 | 0 (0%) | 27 (6.8%) |  | 13 (16.9%) | 191 (32.3%) |  |
| 10 ~ 15 | 0 (0%) | 1 (0.3%) |  | 5 (6.5%) | 55 (9.3%) |  |
| > 15 | 0 (0%) | 0 (0%) |  | 5 (6.5%) | 37 (6.3%) |  |
| ***FIB-4 Score*** | | | | | | |
| < 1.3 | 143 (92.2%) | 348 (87.7%) | 0.46 | 52 (67.5%) | 336 (56.9%) | 0.15 |
| 1.3 ~ 2.67 | 11 (7.1%) | 39 (9.8%) |  | 17 (22.1%) | 144 (24.4%) |  |
| > 2.67 | 1 (0.6%) | 5 (1.3%) |  | 8 (10.4%) | 107 (18.1 %) |  |
| Note: AUD; alcohol use disorder, NAFLD; Non-alcoholic fatty liver disease, FIB-4: Fibrosis-4. | | | | | | |

**Table S4: Median and IQR Analysis of Continuous Variables Shown in Table 1**

|  | **HC**  **(n=155)** | **metHC**  **(n=397)** | **P-Value**  HC vs. metHC | **AUD**  **(n=77)** | **metAUD (n=591)** | **P-Value**  AUD vs. metAUD |
| --- | --- | --- | --- | --- | --- | --- |
| ***Alcohol-Related Characteristics*** | | | | | | |
| Total Drinks (past 90 d) | 24 [41.2] | 15 [40.5] | 0.01 | 418 [748] | 646 [981] | 0.01 |
| No. of Heavy Drinking  Days (past 90 d) | 0 [2] | 0 [1] | 0.21 | 51 [63] | 69 [58] | 0.08 |
| AUDIT-C score | 2 [2] | 2 [3] | 0.08 | 9 [5] | 11 [4] | 0.03 |
| ***Metabolic Criteria*** | | | | | | |
| BMI | 22.4 [2.7] | 27.9 [5.2] | <0.001 | 22.2 [3] | 27.2 [6.2] | <0.001 |
| Serum Glucose, mg/dL | 88 [9] | 91 [11] | <0.001 | 87 [9] | 95 [15] | <0.001 |
| HbA1c, % | 5.1 [0.5] | 5.3 [0.6] | <0.001 | 5.1 [0.6] | 5.3 [0.6] | <0.001 |
| Systolic Blood Pressure,  mm Hg | 113 [13] | 122 [18] | <0.001 | 114 [13] | 131 [20.5] | <0.001 |
| Diastolic Blood  Pressure, mm Hg | 69 [10] | 73 [12] | <0.001 | 72 [12] | 84 [16] | <0.001 |
| Plasma triglycerides,  mg/dL | 60 [33.5] | 73 [47] | <0.001 | 71 [38] | 89 [63.5] | <0.001 |
| LDL cholesterol, mg/dL | 90 [38] | 104 [40.5] | <0.001 | 75 [38] | 92 [45] | <0.001 |
| HDL cholesterol, mg/dL | 67 [21] | 56 [20] | <0.001 | 80 [30] | 62 [21] | <0.001 |
| Total cholesterol, mg/dL | 171 [47.5] | 179 [40] | 0.09 | 168 [47] | 179 [55] | 0.049 |
| ***Chemistry*** | | | | | | |
| Platelet count, K/µL | 244 [60.5] | 240 [81] | 0.43 | 228 [92] | 218 [89.5] | 0.29 |
| GGT, U/L | 15 [8] | 19 [14] | <0.001 | 26 [64] | 49 [100] | <0.001 |
| AST, U/L | 18 [7] | 19 [8] | 0.03 | 26 [21] | 27 [28] | 0.29 |
| ALT, U/L | 13 [8] | 17 [11] | <0.001 | 20 [19] | 26 [24.2] | 0.006 |
| CRP, U/L | 0.6 [0.6] | 1.4 [2.6] | <0.001 | 0.6 [1.1] | 1.8 [3.5] | <0.001 |
| Albumin, g/dL | 4.5 [0.3] | 4.4 [0.4] | <0.001 | 4.2 [0.8] | 4.1 [0.7] | 0.10 |
| Total bilirubin, mg/dL | 0.5 [0.3] | 0.5 [0.3] | 0.004 | 0.6 [0.5] | 0.6 [0.5] | 0.61 |
| Direct bilirubin, mg/dL | 0.1 [0.1] | 0.1 [0] | 0.28 | 0.1 [0.2] | 0.1 [0.2] | 0.17 |
| PT-INR | 1.01 [0.08] | 1.0 [0.1] | 0.08 | 0.98 [0.11] | 0.99 [0.1] | 0.40 |
| ***Liver Fibrosis Scores*** | | | | | | |
| NAFLD Fibrosis Score | -1.01 [1.15] | -0.18 [1.49] | <0.001 | -0.51 [1.64] | 0.70 [2.18] | <0.001 |
| LiverRisk Score | 4.39 [0.82] | 4.88 [0.96] | <0.001 | 5.24 [1.78] | 5.89 [2.71] | <0.001 |
| FIB-4 Score | 0.57 [0.33] | 0.71 [0.50] | <0.001 | 0.94 [0.80] | 1.16 [1.21] | 0.03 |
| ***Psychiatric Scales*** | | | | | | |
| BSA | 0 [1] | 0 [0] | 0.31 | 6 [11] | 9 [12] | 0.003 |
| MADRS | 0 [1] | 0 [1] | 0.77 | 8 [13] | 12 [17.2] | 0.007 |
| STAI-Y2 | 27 [9.5] | 27 [9] | 0.67 | 43 [19] | 46 [20] | 0.22 |
| Note: AUD; alcohol use disorder, AUDIT; The Alcohol Use Disorders Test- Consumption, BMI; body mass index, HbA1c; hemoglobin A1c, LDL cholesterol; low density lipoprotein cholesterol, HDL cholesterol; high density lipoprotein cholesterol, GGT; gamma-glutamyl transferase, ALT; alanine aminotransferase, AST; aspartate aminotransferase, CRP; C-reactive protein, PT-INR; prothrombin time-international normalized ratio, NAFLD; Non-alcoholic fatty liver disease, FIB-4; Fibrosis-4, BSA; Brief Scale for Anxiety, MADRS; Montgomery Asberg Depression Rating Scale, STAI-Y2; Spielberger State-Trait Anxiety Inventory-Y2 Score. | | | | | | |

**Table S5: Prevalence of Psychiatric Disorders by Group,** No. (%)

|  | **HC**  **(n= 155)** | **metHC (n= 397)** | **P-Value**  HC vs metHC | **AUD**  **(n=77)** | **metAUD (n=591)** | **P-Value**  AUD vs metAUD |
| --- | --- | --- | --- | --- | --- | --- |
| ***DSM Psychiatric Disorders*** | | | | | | |
| Bipolar | 0 (0%) | 1 (0.3%) | 1 | 2 (2.6%) | 27 (4.6%) | 0.62 |
| Anxiety | 0 (0%) | 3 (0.8%) | 0.66 | 17 (22.1%) | 187 (31.6%) | 0.11 |
| Depression | 8 (5.2%) | 32 (8.1%) | 0.32 | 38 (49.4%) | 301 (50.9%) | 0.89 |
| PTSD | 3 (1.9%) | 10 (4.0%) | 0.93 | 16 (20.8%) | 158 (26.7%) | 0.33 |
| ADHD/ADD | 1 (0.7%) | 5 (1.3%) | 0.87 | 9 (11.7%) | 77 (13.0%) | 0.88 |
| Eating Disorder | 2 (1.3%) | 5 (1.3%) | 1 | 5 (6.5%) | 23 (3.9%) | 0.44 |
| Agoraphobia and/or  panic disorder | 0 (0%) | 0 (0%) | NA | 1 (1.3%) | 36 (6.1%) | 0.14 |
| Social or specific  phobia | 2 (1.3%) | 8 (2.0%) | 0.057 | 5 (6.5%) | 68 (11.5%) | 0.26 |
| Note: AUD; alcohol use disorder, DSM; Diagnostic and Statistical Manual of Mental Disorders, PTSD; posttraumatic stress disorder, ADHD; attention deficit hyperactivity disorder, ADD; attention deficit disorder. | | | | | | |

**Table S6: Prevalence of Substance Use Disorders by Group,** No. (%)

|  | **HC**  **(n= 155)** | **metHC (n= 397)** | **P-Value**  HC vs metHC | **AUD**  **(n=77)** | **metAUD (n=591)** | **P-Value**  AUD vs metAUD |
| --- | --- | --- | --- | --- | --- | --- |
| ***DSM Substance Use Disorders*** | | | | | | |
| Any DSM illicit drug disorder | 2 (1.3%) | 20 (5.0%) | 0.07 | 48 (62.3%) | 353 (59.1%) | 0.75 |
| Cannabis | 2 (1.3%) | 14 (3.5%) | 0.26 | 32 (41.6%) | 291 (49.2%) | 0.25 |
| Stimulants | 0 (0%) | 5 (1.3%) | 0.37 | 22 (28.6%) | 196 (33.2%) | 0.50 |
| Opioids | 0 (0%) | 9 (2.3%) | 0.13 | 7 (9.1%) | 76 (12.9%) | 0.45 |
| Hallucinogens | 0 (0%) | 1 (0.3%) | 1 | 5 (6.5%) | 55 (9.3%) | 0.53 |
| Sedatives | 0 (0%) | 1 (0.3%) | 1 | 3 (3.9%) | 34 (5.8%) | 0.69 |
| Inhalants | 0 (0%) | 0 (0%) | NA | 0 (0%) | 7 (1.2%) | 0.72 |
| Note: AUD; alcohol use disorder, DSM; Diagnostic and Statistical Manual of Mental Disorders. | | | | | | |

**Figure S2:** **Liver enzymes and CRP values by metabolic severity subgroups in AUD**


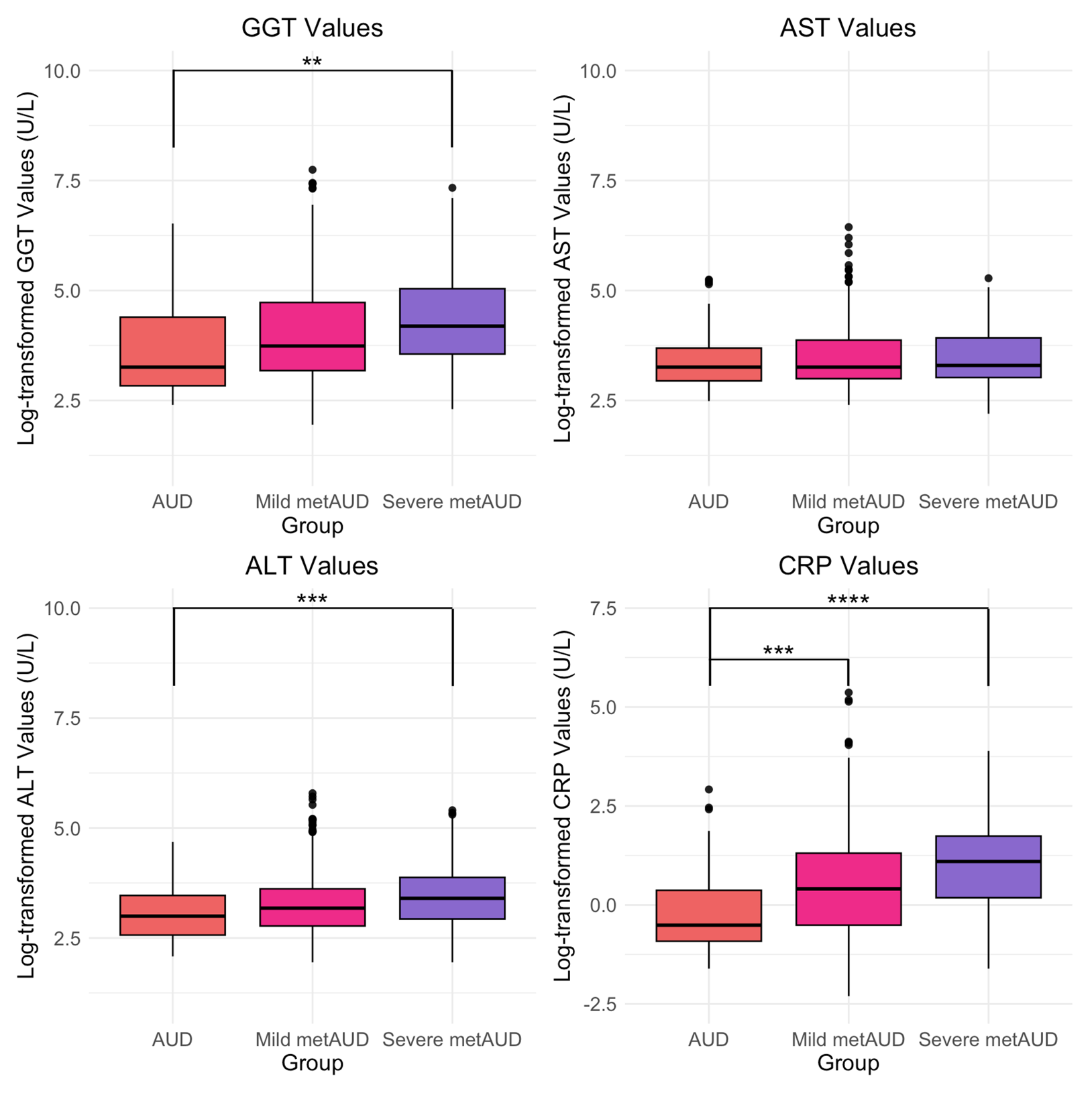


A

C

B

D

**(A)** GGT Values. **(B)** AST Values. **(C)** ALT Values. **(D)** CRP Values. Boxplot shows median and interquartile ranges. Statistical analysis was conducted using ANCOVA, controlling for age, race, and sex with log-transformation. Significance levels are indicated as follows: *p<0.05, **p<0.01, ***p<0.001, ****p<0.0001.

**Table S7: ANCOVA Analysis of Substance Use and Nicotine Use**

|  | **metHC**  **(n=397)** | | | **AUD**  **(n=77)** | | | **metAUD**  **(n=591)** | | |
| --- | --- | --- | --- | --- | --- | --- | --- | --- | --- |
|  | **Effect Size**  (b) | **St. Error** | **P-Value** | **Effect Size**  (b) | **St. Error** | **P-Value** | **Effect Size**  (b) | **St. Error** | **P-Value** |
| ***DSM Substance Use Disorders*** | | | | | | | | | |
| Any DSM Illicit Drug  Abuse or Disorder | 1.13 | 0.75 | 0.13 | 4.59 | 0.75 | <0.001 | 4.33 | 0.72 | <0.001 |
| Cannabis Use | 0.81 | 0.77 | 0.29 | 4.25 | 0.75 | <0.001 | 4.00 | 0.72 | <0.001 |
| ***Nicotine Use*** |  |  |  |  |  |  |  |  |  |
| Current Smoker | 0.92 | 0.63 | 0.14 | 4.04 | 0.63 | <0.001 | 3.67 | 0.60 | <0.001 |
| FTND Score | 0.73 | 1.47 | 0.62 | 1.09 | 1.43 | 0.44 | 1.75 | 1.39 | 0.21 |
| Note: AUD; alcohol use disorder, DSM; Diagnostic and Statistical Manual of Mental Disorders, FTND; Fagerström Test for Nicotine Dependence. | | | | | | | | | |

**Table S8: Sensitivity Analysis of FIB-4 Scores using Ages 35-65**

|  | **metHC**  **(n=191)** | | | **AUD**  **(n=42)** | | | **metAUD**  **(n=423)** | | |
| --- | --- | --- | --- | --- | --- | --- | --- | --- | --- |
|  | **Effect Size**  (b) | **St. Error** | **P-Value** | **Effect Size**  (b) | **St. Error** | **P-Value** | **Effect Size**  (b) | **St. Error** | **P-Value** |
| ***FIB-4 Score (Ages 35- 65)*** | 0.14 | 0.60 | 0.81 | 1.40 | 0.76 | 0.07 | 1.53 | 0.57 | 0.008 |
| Note: AUD; alcohol use disorder. FIB-4: Fibrosis-4. | | | | | | | | | |

**Table S9:** **Prevalence of** **Psychiatric Disorders by Metabolic Dysfunction Severity in AUD,** No. (%)

|  | **Mild metAUD**  **(n = 390)** | **Severe metAUD**  **(n = 201)** | **P-Value** |
| --- | --- | --- | --- |
| ***DSM Psychiatric Disorder*** | | | |
| Bipolar | 16 (4.1%) | 11 (5.5%) | 0.58 |
| Anxiety | 118 (30.3%) | 69 (34.3%) | 0.36 |
| Depression | 197 (50.5%) | 104 (51.7%) | 0.84 |
| PTSD | 103 (26.4%) | 55 (27.4%) | 0.88 |
| ADHD/ADD | 57 (14.6%) | 20 (10.0%) | 0.14 |
| Eating Disorder | 16 (4.1%) | 7 (3.5%) | 0.88 |
| Agoraphobia and/or panic disorder | 24 (6.2%) | 12 (6.0%) | 1 |
| Social or specific phobia | 45 (11.5%) | 23 (11.4%) | 1 |
| Note: AUD; alcohol use disorder, DSM; Diagnostic and Statistical Manual of Mental Disorders, PTSD; posttraumatic stress disorder, ADHD; attention deficit hyperactivity disorder, ADD; attention deficit disorder. | | | |

**Table S10: FibroScan Analysis**

|  | **AUD**  **(n=3)** | **Mild metAUD**  **(n=42)** | **Severe metAUD**  **(n=17)** | **P-Value**  AUD vs mild metAUD | **P-Value**  AUD vs severe metAUD | **P-Value**  Mild metAUD vs severe metAUD |
| --- | --- | --- | --- | --- | --- | --- |
| ***FibroScan Data,*** mean (SD) | | | | | | |
| CAP Score, dB/m | 185 (42.2) | 244 (54.5) | 288 (52.9) | 0.13 | 0.03 | 0.007 |
| Liver Stiffness, kPa | 4.57 (0.76) | 6.24 (3.78) | 15.2 (21.4) | 0.04 | 0.056 | 0.10 |
| ***FibroScan Data,*** median [IQR] | | | | | | |
| CAP Score, dB/m | 204 [39] | 232 [72] | 264 [98] | 0.08 | 0.004 | 0.004 |
| Liver Stiffness, kPa | 4.9 [0.70] | 5.3 [2.9] | 7.1 [5.5] | 0.45 | 0.07 | 0.07 |
| Note: AUD; alcohol use disorder, CAP; controlled attenuation parameter. | | | | | | |

**Figure S3: Exploratory analysis of FibroScan liver stiffness scores by metabolic severity subgroups in AUD**


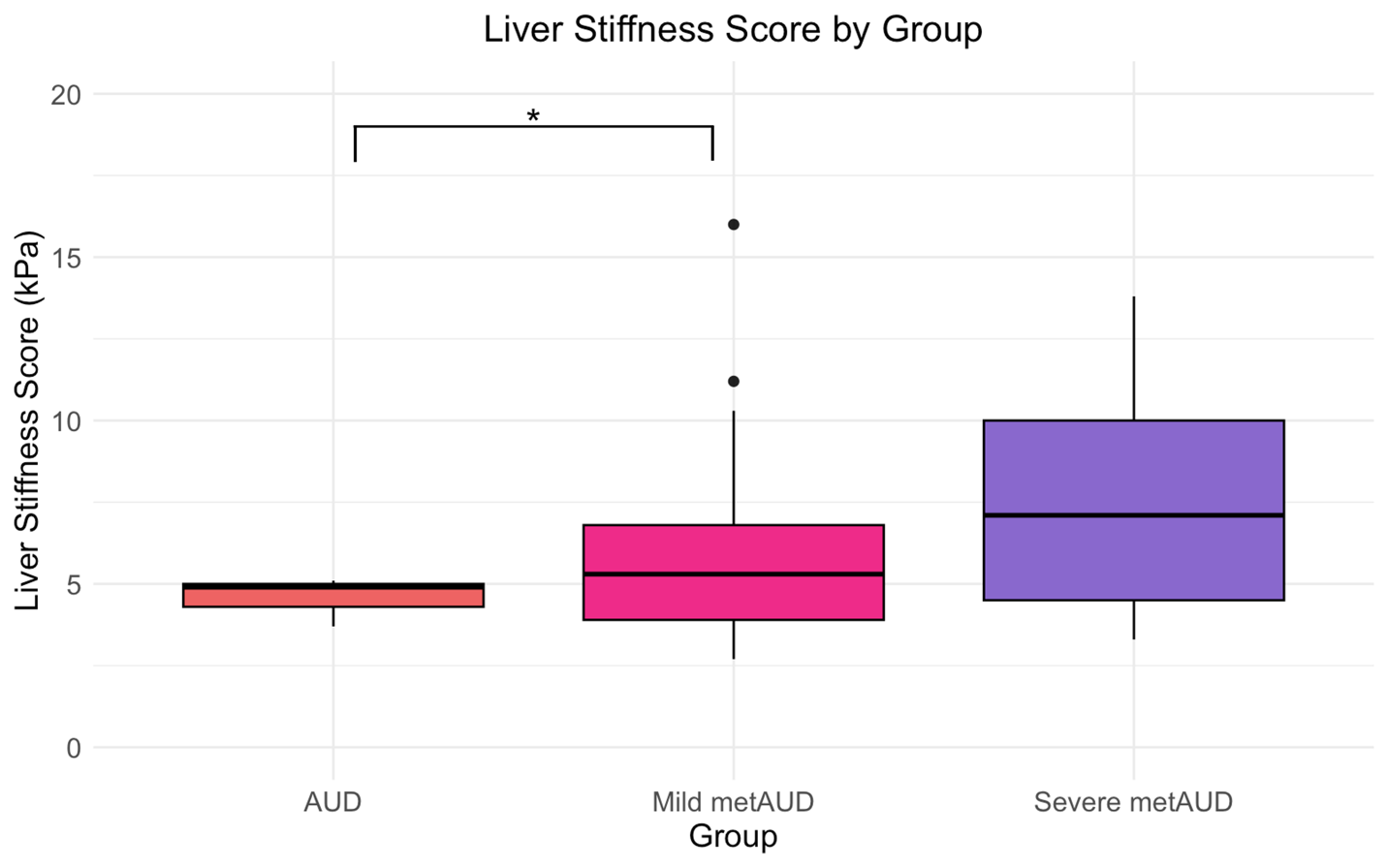


Boxplot shows median and interquartile ranges. Statistical analysis was conducted using t-tests. Significance levels are indicated as follows: *p<0.05, **p<0.01, ***p<0.001, ****p<0.0001. For FibroScan data, sample sizes include AUD=3, mild metAUD=42, and severe metAUD=17.
